# Effect of different folic acid doses on methotrexate-related toxicity and its association with erythrocyte methotrexate-polyglutamates in patients with rheumatic diseases: a single-center exploratory randomized controlled trial

**DOI:** 10.1007/s10067-026-08195-8

**Published:** 2026-06-06

**Authors:** Yusuke Yoshida, Yohei Hosokawa, Takanori Taogoshi, Naoya Oka, Hiroki Kobayashi, Genki Kidoguchi, Kei Araki, Michinori Ishitoku, Tomohiro Sugimoto, Sho Mokuda, Hiroaki Matsuo, Shintaro Hirata

**Affiliations:** 1 Department of Clinical Immunology and Rheumatology, Hiroshima University Hospital, 1-2-3 Kasumi Minami, Hiroshima, 734-8551 Japan; 2 Department of Rheumatology, JA Hiroshima General Hospital, Hatsukaichi, Japan; 3 Department of Pharmaceutical Services, Hiroshima University Hospital, Hiroshima, Japan; 4 Department of Rheumatology, Hiroshima Prefectural Hospital Organization Futabanosato Prefectural Hospital, Hiroshima, Japan; 5 Division of Laboratory Medicine, Hiroshima University Hospital, Hiroshima, Japan

**Keywords:** Fatigue, Folic acid, Hepatotoxicity, Methotrexate, Polyglutamated methotrexate, Rheumatoid arthritis

## Abstract

**Introduction:**

Folic acid is routinely co-administered with methotrexate (MTX) to reduce toxicity; however, the optimal dose remains unclear. This study investigated whether increasing the weekly folic acid dose from 5 to 10 mg reduces MTX-related toxicity in patients receiving stable-dose MTX therapy.

**Methods:**

In this single-center, open-label, randomized controlled trial, 44 patients with rheumatic diseases on stable-dose MTX either received 10 mg (ARM-1) or 5 mg (ARM-2) of folic acid per week for 12 weeks. Liver damage, other MTX-related toxicities, and erythrocyte MTX-polyglutamate (MTX-PG) concentrations were compared between groups using logistic regression analysis or analysis of covariance. Japan Registry of Clinical Trials (number: jRCT1061230085).

**Results:**

Forty-two patients completed the study. The presence of liver damage at Day 84 did not differ significantly between the two groups (OR 0.41, 95% CI, 0.01–5.75, *p* = 0.506). Adjusted mean differences in aspartate transaminase and alanine transaminase at Day 84 between the groups were − 0.33 U/L (95% CI, − 2.68 to 2.02; *p* = 0.779) and − 0.44 U/L (95% CI, − 5.39 to 4.51; *p* = 0.859). Comparison of outcomes between groups at Day 84 showed no significant differences in any outcomes, except for fatigue severity (adjusted mean difference, − 0.87; 95% CI, − 1.71 to − 0.04; *p* = 0.041). Baseline fatigue was associated with baseline MTX-PG2 or MTX-PG1-2 concentrations (OR, 1.06; *p* = 0.049 or OR, 1.03; *p* = 0.040, respectively).

**Conclusions:**

Increasing folic acid dose to 10 mg/week showed little effect on reducing MTX-related toxicity, although it may improve fatigue in selected patients.

**Table:** 

**Key Points** • *Increasing the weekly folic acid dose from 5 mg to 10 mg showed no additional benefit in reducing methotrexate-related toxicity in patients on stable-dose MTX.* • *Higher-dose folic acid was associated with improved fatigue severity, indicating a potential therapeutic option for patients who develop fatigue.*

**Supplementary Information:**

The online version contains supplementary material available at 10.1007/s10067-026-08195-8.

## Introduction

Methotrexate (MTX) is widely used as the standard treatment for rheumatic diseases and is considered the first-line treatment for rheumatoid arthritis (RA) because of its favorable balance of efficacy, safety, and cost-effectiveness [1]. However, some patients experience fatigue, oral stomatitis, nausea, bone marrow suppression, and hepatotoxicity during MTX therapy. Therefore, supplementation of low-dose of folic acid is recommended to reduce MTX-related toxicity and to improve treatment adherence [1–5]. In Japan, the co-administration of folic acid at a dose of 5 mg per week, taken 24–48 h after the most recent MTX dose, is officially recommended in practical guidelines for RA management [6, 7]. However, few studies have investigated the effects of different folic acid doses on MTX-related toxicity [8–11]; therefore, the optimal folic acid regimen remains debatable.

To understand the potential effect of folic acid on MTX efficacy and toxicity, it is important to consider the mechanism of action of MTX. After administration, MTX is transported into cells via reduced folate carrier 1 and is rapidly cleared from circulation. Within cells, MTX initially exists in its monoglutamated form (MTX-PG1), which is considered biologically less active. Subsequently, it is converted by folylpolyglutamate synthetase (FPGS) into MTX-PG2 and longer-chain polyglutamated forms (MTX-PG3-PGn) that are primarily responsible for the major immunomodulatory effects of MTX [12]. Among the anti-inflammatory mechanisms of MTX-PGs, two pathways are considered central: inhibition of dihydrofolate reductase, which alters folate-dependent pathways in inflammatory cells, and inhibition of aminoimidazole-4-carboxamide ribonucleotide (AICAR) transformylase, which leads to intracellular AICAR accumulation and enhanced adenosine release [12, 13]. The concentration of MTX-PGs in erythrocytes can be quantified as a useful biomarker for evaluating the efficacy and hepatotoxicity of MTX [14, 15]. However, longitudinal evaluations of erythrocyte MTX-PG concentrations during stable-dose MTX therapy have not been reported.

In this study, we aimed to investigate the effects of two different doses of folic acid on the safety and efficacy of MTX in patients with rheumatic diseases. We compared the changes in erythrocyte MTX-PG concentrations over the study period between the two groups and explored the associations between clinical outcomes and MTX-PG concentrations.

## Materials and methods

### Study design and participants

This single-center, parallel-group, open-label randomized controlled trial (RCT) was conducted at Hiroshima University Hospital, Japan. In the trial, patients with rheumatic diseases receiving stable MTX therapy were assigned to one of two folic acid supplementation regimens, and the effects on MTX toxicity and disease activities of the underlying rheumatic diseases were compared (Supplementary Fig. 1).

Patients were eligible if they: (1) were ≥ 18 years at the time of consent, (2) were diagnosed with rheumatic diseases more than 12 weeks prior and were receiving stable doses of oral MTX for more than 8 weeks, (3) were taking folic acid 5 mg per week 24–48 h after the most recent MTX dose, or (4) whose written consents for participation were obtained. The rheumatic diseases include RA, psoriatic arthritis, and others. In addition, patients who fulfilled the following criteria were excluded from the trial: (1) had regularly taken supplements or health foods containing folic acid within 8 weeks before starting the study, (2) demonstrated poor medication adherence, (3) were unable to attend study visits during the 12-week study period, or (4) were deemed ineligible by the principal investigator or sub-investigator.

This trial was approved by the Hiroshima University Certified Review Board in accordance with the Declaration of Helsinki (number: CRB2023-0006) and registered in the Japan Registry of Clinical Trials (number: jRCT1061230085; date of registration: December 25, 2023). Written consent was obtained from all participants.

### Interventions

From baseline (Day 1) to Day 84, participants in the ARM-1 group received folic acid at 10 mg per week (5 mg × 2 tablets), administered 24 h after the most recent MTX dose. Participants in the ARM-2 group continued folic acid at 5 mg per week (1 tablet), administered 24 h after the most recent MTX intake. The 10 mg per week folic acid dosage was chosen as the increased-dose regimen for comparison with the standard 5 mg per week regimen considering the relatively low average dose of MTX used for Japanese patients [6, 7]. Participants continued the same dose of MTX during the study period unless severe adverse events or disease flare occurred. Visits scheduled for Day 84 were allowed within 2 weeks.

### Primary and secondary outcomes

The primary outcome was the difference between the ARM-1 and ARM-2 groups in the presence of liver damage at Day 84, defined as aspartate transaminase (AST) > 34 U/L or alanine transaminase (ALT) > 42 U/L. Changes in liver transaminase levels from baseline to Day 84 were assessed as supportive analyses. Additional outcomes included: (1) differences in changes in patient-reported outcomes (PROs), laboratory markers, and composite measures of RA; and (2) differences in changes in erythrocyte MTX-PG concentrations between the two groups.

### Randomization and allocation

Enrolled patients were randomized into the ARM-1 or ARM-2 groups at a ratio of 1:1. Randomization was stratified by weekly dose of MTX (≥ 10 mg per week or < 10 mg per week), kidney function (eGFR ≥ 60 mL/min/1.73 m^2^ or < 60 mL/min/1.73 m^2^) and body mass index (BMI) (BMI < 18.5 kg/m^2^, 18.5 ≤ BMI < 25.0 kg/m^2^ or 25.5 ≤ 60 BMI) using research electronic data capture (REDCap) [16, 17].

### Laboratory and clinical assessment

Clinical information was collected using REDCap [16, 17]. PROs included gastrointestinal symptoms, oral ulcers, and fatigue. Laboratory markers included complete blood count, mean corpuscular volume (MCV), AST, ALT, C-reactive protein (CRP), erythrocyte sedimentation rate (ESR), and MTX-PG1-7 in erythrocytes. For patients with RA, clinical data regarding the Healthcare Assessment Questionnaire Disability Index (HAQ-DI), composite measures with the Clinical Disease Activity Index (CDAI) [18], and Simplified Disease Activity Index (SDAI) [19] were obtained. The severity of general fatigue during the previous week was assessed using a numerical rating scale (0–10).

### Measurements of erythrocyte MTX-PG concentrations

Whole blood samples were obtained from patients in EDTA-2Na tubes and centrifuged. Erythrocyte samples were purified and stored in − 80-degree refrigerator. After destroying the erythrocytes, samples were extracted using Oasis WAX 1 cc Extraction Cartridges (Waters, Milford, MA, USA). The concentrations of individual MTX-PGs in erythrocytes were measured using liquid chromatography-tandem mass spectrometry (Shimadzu Corporation, Kyoto, Japan) following the methods described in a previous study [20]. MTX-PG1-7 standards were purchased from Schirmer Laboratories (Jona, Switzerland).

### Statistical analyses

Baseline characteristics were summarized as median (interquartile range) or number (%). Associations between baseline MTX-PG concentrations and MTX doses or fatigue symptom were evaluated using the Spearman’s rank correlation coefficient or logistic regression analysis. Longitudinal binary outcomes at Day 84 were compared between groups using logistic regression models adjusted for baseline values of the corresponding outcomes. Odds ratios (ORs) and 95% confidence intervals (CIs) were estimated using likelihood-based methods. Continuous outcomes were compared between groups at Day 84 using an analysis of covariance (ANCOVA) adjusted for baseline values of the corresponding outcomes. Results are presented as differences in adjusted means, least-squares means (LSMs), with 95% CIs, and *p*-values. LSM changes were derived from the ANCOVA model assuming a baseline value equal to the overall mean. As 42 of 44 participants completed the study and no missing data were present among completers, complete-case analyses were performed to compare longitudinal changes in adverse events and laboratory or disease-related parameters within and between the two groups. Statistical significance was set at *p* < 0.05. All statistical analyses were performed using JMP Student Edition 18.2.1 (SAS Institute Inc, USA), and figures were drawn using Prism 10.0.2 (GraphPad, USA).

## Results

### Patient characteristics at baseline and follow-ups

**Fig. 1:**
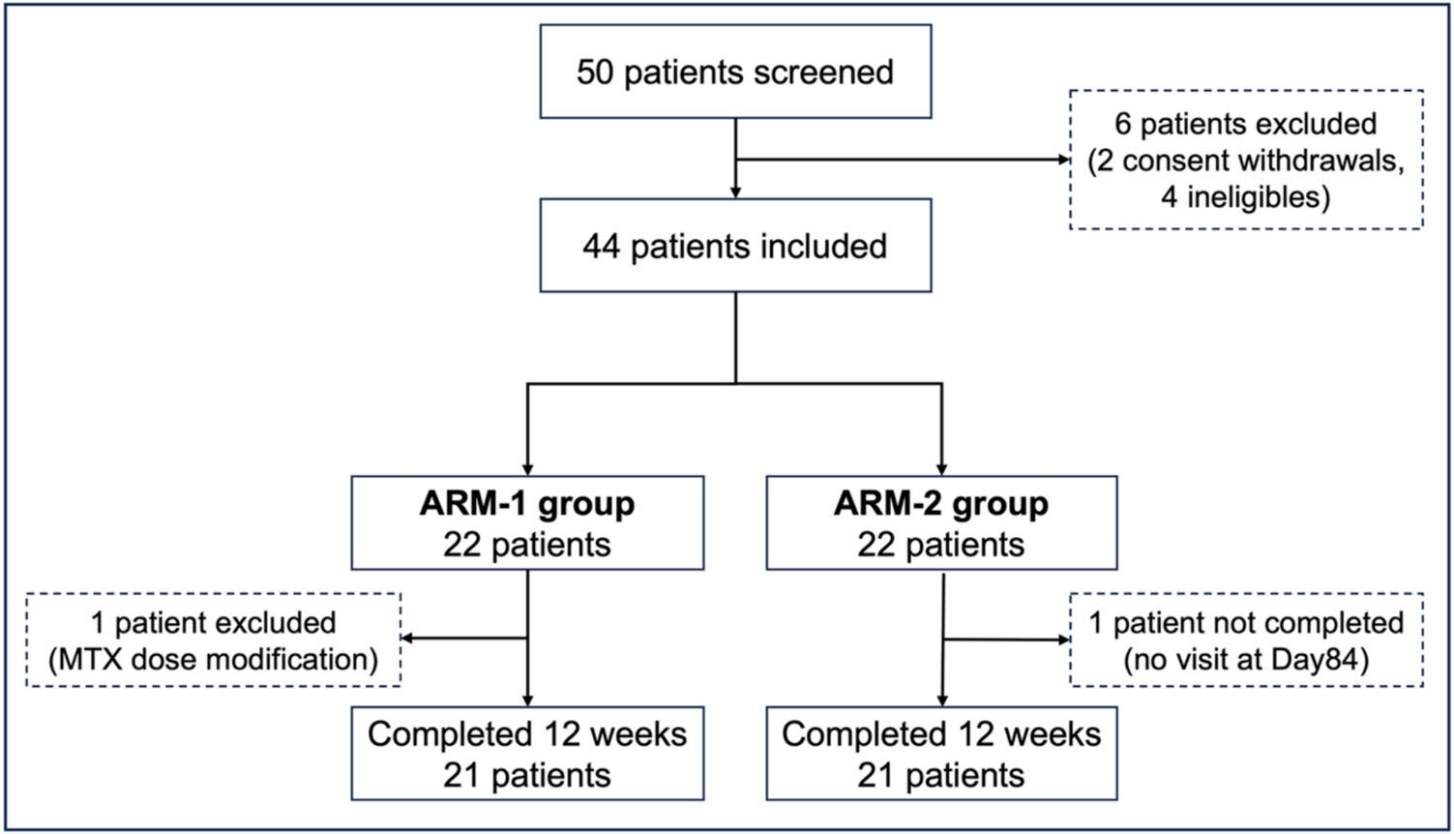
Flow chart of study participants. Co-administration of folic acid (10 mg/week in ARM-1 and 5 mg/week in ARM-2) during MTX therapy. MTX, methotrexate

This study enrolled 50 Japanese patients with rheumatic disease who were receiving a stable dose of MTX. Six patients were excluded before randomization due to withdrawal (*n* = 2) and ineligibility (*n* = 4), as shown in Fig. 1. Among the remaining 44 patients, 22 (including 18 with RA) were randomized into the ARM-1 group and 22 (including 19 with RA) to ARM-2 group. The median age was 65.5 vs. 61.5 years old, with a weekly dose of MTX being 8 mg vs. 8 mg, respectively (Table 1). The details of MTX administration, including the weekly MTX dose, dosing regimen (split or single), and timing of folic acid administration after the last MTX intake, are shown in Supplementary Fig. 2a–c. MTX-PG1-5 was detectable; however, the MTX-PG6 and MTX-PG7 concentrations were below the limit of quantification in all patients. MTX-PG concentrations were well correlated with the weekly dose of MTX for MTX-PG3 (rho = 0.69, *p* < 0.001), MTX-PG4 (rho = 0.67, *p* < 0.001), and MTX-PG5 (rho = 0.48, *p* = 0.001), but not for MTX-PG1, and were weakly correlated with MTX-PG2 (rho = 0.06, *p* = 0.680, and rho = 0.30, *p* = 0.049, respectively) (Supplementary Fig. 2 d).

**Table 1:** Baseline characteristics of enrolled patients

	**ARM-1 (10 mg folic acid),** ***N* = 22**	**ARM-2 (5 mg folic acid),** ***N* = 22**	***p***
Age (yrs)	65.5 (50–76.5)	61.5 (52–70.8)	0.597
Female	16 (72.7)	16 (72.7)	1.000
Body mass index	22.6 (20.3–24.7)	22.6 (19.7–24.6)	0.760
Serum creatinine (mg/dL)	0.70 (0.61–0.89)	0.68 (0.54–0.81)	0.342
Underlying disease			
Rheumatoid arthritis	18 (81.8)	19 (86.4)	
Psoriatic arthritis	1 (4.6)	2 (9.1)	
Others^†^	3 (13.6)	1 (4.6)	
Treatment			
Use of MTX	22 (100)	22 (100)	1.000
Weekly dose of MTX (mg)	8 (4–12)	8 (6–10)	0.721
Glucocorticoid use	3 (13.6)	7 (31.8)	0.150
PSL equivalent dose (mg)	2 (2–2.5)	3 (2–5)	0.203
Use of Biologics	6 (27.3)	5 (22.7)	0.728
Use of TNF inhibitors	4	5	
Use of Others	2	0	
Symptoms of MTX-related toxicity			
Oral ulcers	4 (18.2)	1 (4.6)	0.154
Gastrointestinal symptoms	3 (13.6)	1 (4.6)	0.294
General fatigue (presence)	15 (68.2)	12 (54.6)	0.353
General fatigue (severity)	1.5 (0–1.5)	1 (0–4)	0.689
Disease activity markers			
CRP (mg/dL)	0.07 (0.02–0.31)	0.08 (0.02–0.20)	0.646
ESR (mm/h)	21 (11.5–30)	15 (8–20)	0.166
CDAI in RA^‡^	1.4 (0.1–6.3)	4.7 (0.2–9.2)	0.075
SDAI in RA^‡^	1.9 (0.1–6.3)	4.7 (0.2–9.2)	0.100
HAQ-DI in RA^‡^	0 (0–0.38)	0.5 (0–1)	0.253

Variables are described as number (percentage) or median (interquartile range)

Comparison of the data between the ARM-1 and ARM-2 groups was made by Wilcoxon rank sum test or chi-square test

^†^One with granulomatosis with polyangiitis, 1 with adult-onset Still’s disease and 1 with giant cell arteritis in the ARM-1 group, and 1 with juvenile idiopathic arteritis in the ARM-2 group

^‡^18 in the ARM-1 group and 19 in the ARM-2 group

Abbreviations: MTX, methotrexate; PSL, prednisolone; TNF, tumor necrotizing factor; CRP, C-reactive protein; ESR, erythrocyte sedimentation rate; CDAI, clinical disease activity index; SDAI, simplified disease activity index; HAQ-DI, health assessment questionnaire disability index

During follow-up, one patient with ARM-1 changed the route of MTX administration from oral to subcutaneous, and one patient with ARM-2 dropped out of the study due to missed visits. For the longitudinal analyses, the remaining 42 patients completed the study while maintaining their initial MTX dose. Adherence to MTX and folic acid was confirmed in the interview on Day 84.

### Differences in liver damage at Day 84 between the ARM groups

**Fig. 2:**
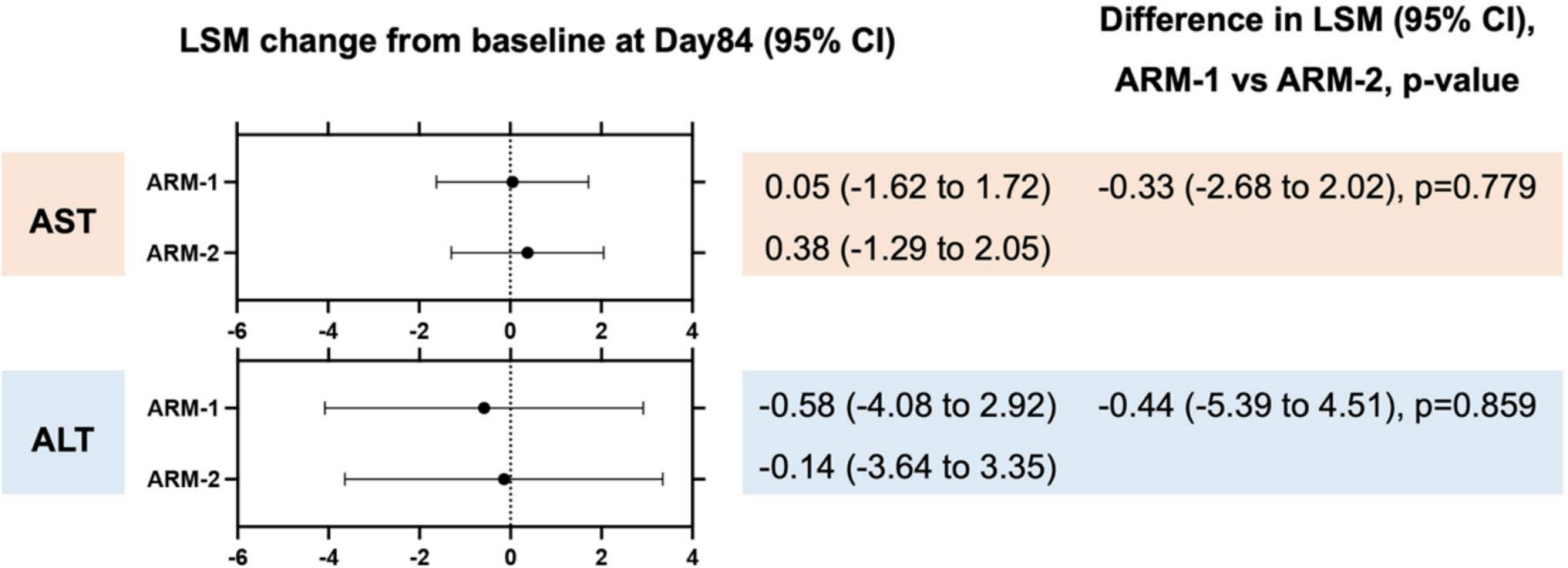
Differences in changes in liver damage between groups at Day 84. Differences in LSM changes in AST and ALT levels from baseline at Day 84 between the ARM-1 (10 mg per week folic acid) and ARM-2 (5 mg per week folic acid) groups. LSM, least-squares mean; AST, aspartate transaminase; ALT, alanine transaminase

Liver damage was observed in three (14.3%) patients at baseline and one (4.8%) at Day 84 in the ARM-1 group, and in three (14.3%) at baseline and two (9.5%) at Day 84 in the ARM-2 group. The presence of liver damage at Day 84 did not differ significantly between the groups (OR, 0.41; 95% CI, 0.01 to 5.75; *p* = 0.506). In addition, the LSM changes from baseline to Day 84 in AST and ALT were 0.05 U/L (95% CI, − 1.62 to 1.72) and − 0.58 U/L (95% CI, − 4.08 to 2.92), respectively, in ARM-1, and 0.38 U/L (95% CI, − 1.29 to 2.05) and − 0.14 U/L (95% CI, − 3.64 to 3.35), respectively, in ARM-2. The adjusted mean differences between the groups at Day 84 were − 0.33 U/L (95% CI, − 2.68 to 2.02; *p* = 0.779) for AST and − 0.44 (95% CI, − 5.39 to 4.51; *p* = 0.859) U/L for ALT (Fig. 2).

### Differences in MTX-related toxicity and clinical efficacy of MTX between groups

When outcomes were compared between groups at Day 84, no significant differences were observed in MCV, CRP, ESR, CDAI, SDAI, or HAQ-DI. In contrast, changes in fatigue severity were significant in ARM-1 compared to ARM-2 (adjusted mean difference, − 0.87; 95% CI, − 1.71 to − 0.04; *p* = 0.041) (Table 2).

**Table 2:** Differences in changes in outcomes at Day 84 adjusted for the baseline values between groups

	**N**	**Day 84 Mean** **(SD)**	**LSM change from baseline at Day 84 (95% CI)**	**Difference in LSM (95% CI), ARM-1 vs ARM-2, *p*-value**
MCV (fL)				
ARM-1	21	96.01 (5.44)	0.58 (− 0.48 to 1.64)	0.34 (− 1.17 to 1.85), *p* = 0.656
ARM-2	21	93.76 (5.51)	0.25 (− 0.82 to 1.31)	
CRP (mg/dL)				
ARM-1	21	0.20 (0.30)	− 0.03 (− 0.11 to 0.07)	0.04 (− 0.08 to 0.17), *p* = 0.480
ARM-2	21	0.11 (0.15)	− 0.07 (− 0.16 to 0.03)	
ESR (mm/h)				
ARM-1	21	23.19 (14.09)	2.75 (− 1.51 to 7.02)	3.71 (− 2.36 to 9.77), *p* = 0.224
ARM-2	21	18.10 (13.19)	− 0.95 (− 5.22 to 3.31)	
CDAI				
ARM-1	18	2.75 (3.88)	− 0.25 (− 1.17 to 0.67)	− 0.29 (− 1.58 to 1.01), *p* = 0.655
ARM-2	19	6.41 (7.18)	0.04 (− 0.83 to 0.91)	
SDAI				
ARM-1	18	2.96 (3.85)	− 0.22 (− 1.08 to 0.66)	− 0.19 (− 1.41 to 1.03), *p* = 0.754
ARM-2	19	6.47 (7.22)	− 0.03 (− 0.84 to 0.80)	
HAQ-DI				
ARM-1	18	0.21 (0.48)	− 0.05 (− 0.14 to 0.05)	− 0.02 (− 0.14 to 0.10), *p* = 0.787
ARM-2	19	0.47 (0.57)	− 0.03 (− 0.12 to 0.06)	
Fatigue (severity)				
ARM-1	21	0.95 (1.40)	− 1.2 (− 1.79 to − 0.61)	− 0.87 (− 1.71 to − 0.04), *p* = 0.041
ARM-2	21	1.76 (1.95)	− 0.33 (− 0.92 to 0.26)	
Oral ulcers (times)				
ARM-1	21	0.19 (0.40)	0.04 (− 0.08 to 0.17)	0.17 (− 0.001 to 0.35), *p* = 0.051
ARM-2	21	0 (0)	− 0.13 (− 0.25 to − 0.01)	

*SD* standard deviation, *LSM* least-squares mean, *CI* confidence interval, *MCV* mean corpuscular volume, *CRP* C-reactive protein, *ESR* erythrocyte sedimentation rate, *CDAI* Clinical Disease Activity Index, *SDAI* Simplified Disease Activity Index, *HAQ-DI* Healthcare Assessment Questionnaire Disability Index

### Adverse events at any time of this trial

The adverse events, irrespective of MTX toxicity, are summarized in Supplementary Table 1. No severe adverse events were observed during the study period. The most frequent adverse event was acute upper respiratory infection, which occurred in two patients with ARM-1 and three patients with ARM-2.

### Changes in MTX-PG concentrations in each ARM group

**Fig. 3:**
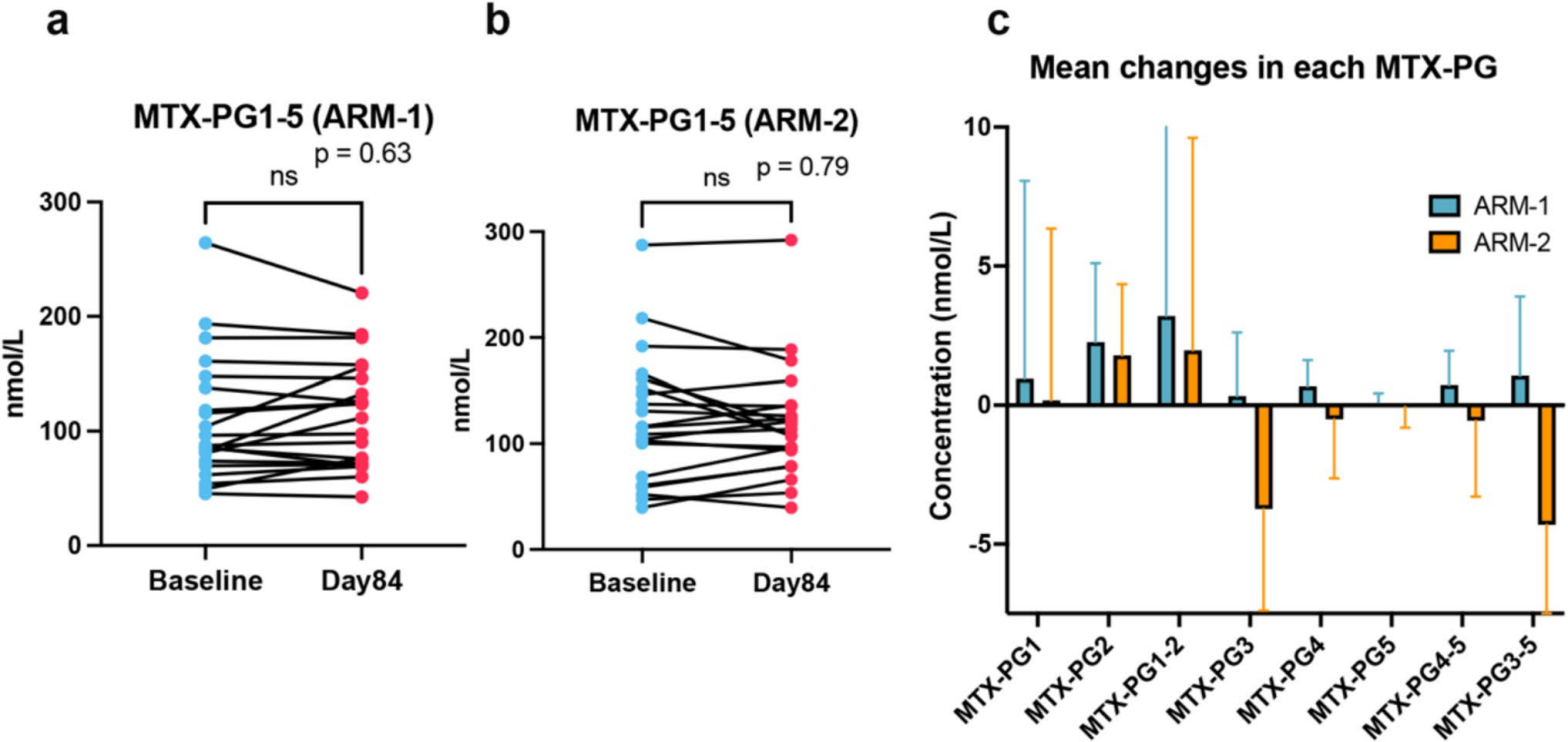
MTX-PG concentrations over 12 weeks in the two ARM groups. Longitudinal changes in total and individual MTX-PG concentrations over 12 weeks in ARM-1 (10 mg per week folic acid) and ARM-2 (5 mg per week folic acid). **a**, **b** Changes in total MTX-PG1-5 concentration in each ARM group over the 12-week period. **c** Mean change in individual MTX-PG concentrations during the same period. MTX-PG, methotrexate-polyglutamate

Erythrocyte MTX-PG concentration in both ARM groups showed no significant changes over time in total MTX-PG1-5 concentrations (Fig. 3a, b), whereas the mean concentrations of individual MTX-PG varied despite a stable dose of MTX (Fig. 3c). Comparison of MTX-PG concentrations between groups at Day 84 showed no significant differences (Supplementary Table 1). We further evaluated the association of general fatigue with MTX-PG concentrations at baseline. Baseline MTX-PG2 and MTX-PG1-2 concentrations were associated with baseline fatigue presence (OR, 1.06; *p* = 0.049 and OR, 1.03; *p* = 0.040, respectively) (Table 3).

**Table 3:** Association of baseline erythrocyte MTX-PG concentrations with fatigue presence (*N* = 44)

	**Odds ratio**	**95% CI**	***p***	**AUC**	**Cut-off**	**Sensitivity**	**Specificity**
MTX-PG1	1.03	1.00 to 1.08	0.067	0.62	46.8	33.3	93.8
MTX-PG2	1.06	1.00 to 1.14	**0.049**	0.69	19.5	85.2	50.0
MTX-PG1-2	1.03	1.00 to 1.06	**0.040**	0.66	53.7	70.4	62.5
MTX-PG3	1.02	1.00 to 1.06	0.101	0.69	39.3	63.0	75.0
MTX-PG4	1.01	0.96 to 1.08	0.638	0.63	9.2	48.2	81.3
MTX-PG5	0.99	0.86 to 1.14	0.820	0.42	13.6	100	6.3
MTX-PG3-5	1.01	0.99 to 1.03	0.258	0.68	45.3	63.0	75.0
MTX-PG4-5	1.00	0.97 to 1.05	0.779	0.56	9.5	45.5	76.2
MTX-PG1-5	1.01	1.00 to 1.03	0.066	0.72	118.3	55.6	87.5

*MTX-PG* methotrexate-polyglutamate, *CI* confidence interval, *AUC* area under curve

## Discussion

In this study, we found that neither the presence of liver damage nor the levels of liver transaminases differed between the groups receiving 10 mg and 5 mg per day of folic acid in patients treated with a stable dose of MTX, although fatigue improved in the 10-mg group. Changes in total or individual MTX-PG concentrations did not differ between groups over the study period. At baseline, only long-chain MTX-PGs (MTX-PG3-5) were correlated with MTX dose, whereas fatigue was associated with short-chain MTX-PGs (MTX-PG1-2). Therefore, the routine use of 10 mg folic acid does not appear to be an effective approach to prevent hepatotoxicity, oral ulcers, and gastrointestinal symptoms. Moreover, increasing the folic acid dose from 5 to 10 mg per day may not be effective in reducing MTX-related liver damage.

To our knowledge, this is the first RCT to evaluate MTX-related toxicity under a stable MTX dose when co-administered with weekly folic acid at doses of 10 mg and 5 mg while simultaneously assessing erythrocyte MTX-PG concentrations. Our main results were consistent with those of three previous RCTs, which found that different doses of folic acid during MTX therapy did not affect MTX toxicity or efficacy. Of them, folic acid doses of 27.5 mg vs. 5 mg per week, 30 mg vs. 10 mg per week, and 5 mg vs. 0.8 mg per week were compared in patients with RA [8–10] (Supplementary Table 3). Notably, the timing of folic acid administration varied across trials. In two trials, folic acid was taken on 5 or 6 days per week when MTX was not administered, which differs from the current recommendation of taking folic acid at least 24 h after MTX in one or two doses [21]. In this context, our study provides additional insights into the optimization of folic acid supplementation during MTX therapy in patients with rheumatic diseases.

In our study, the concentrations of long-chain MTX-PG, but not short-chain MTX-PG, were closely associated with the weekly MTX dose, which is consistent with previous findings [22]. Short-chain MTX-PGs remain detectable at very low MTX doses, and their concentrations may be influenced by the timing of blood sampling relative to MTX and folic acid administration. Notably, fatigue was associated with short-chain MTX-PGs and was the only MTX-related toxicity that improved in our study with an increased dose of folic acid. This finding suggests that patients with altered intracellular polyglutamation of MTX may be more susceptible to fatigue. Yamamoto et al. reported that polymorphisms in *FPGS*, a key gene coding an enzyme involved in intracellular MTX polyglutamation, were associated with MTX-related toxicity and MTX-PG3-5/1–2 ratios [23]. Taken together, these findings raise the possibility that patients with a reduced capacity to form long-chain MTX-PGs could benefit from increased folic acid supplementation.

One strength of our study is that all patients included in the analyses received a stable dose of MTX for at least 12 weeks before and after enrollment. Moreover, the randomization process was stratified by MTX dose, kidney function, and BMI, and the timing of folic acid administration was strictly standardized. These methodological features ensured the comparability between the two groups and enhanced the robustness of our evaluation. In addition, we prospectively collected PROs related to MTX toxicity using standardized questionnaires at each visit, enabling a more accurate assessment of subjective adverse events, which are often underreported in retrospective studies.

Despite these strengths, the study had some limitations that should be considered. First, the sample size was relatively small and was determined based on the maximum number of patients feasible at a single center, which was considered acceptable because the study was exploratory in nature. Second, because the incidence of hepatotoxicity was low and the confidence intervals were wide, clinically meaningful differences between the two regimens cannot be excluded. A substantially larger non-inferiority trial would likely be required to determine whether the safety of 5 mg folic acid supplementation is comparable to that of 10 mg supplementation. Third, the intervention was not blinded, and subjective outcomes such as fatigue may have been susceptible to placebo effects. However, the association between fatigue and objectively measured MTX-PG profiles supports the biological plausibility of our findings. Fourth, although most participants had RA, a small number of patients with other rheumatic diseases were also included, but as the number of non-RA patients was limited, the influence of disease heterogeneity on MTX-related toxicity and MTX-PG concentrations could not be fully evaluated. Fifth, our study population received lower MTX doses than those commonly prescribed in other countries, which may limit the applicability of our findings to patients receiving higher MTX doses. Japanese patients may be more prone to intolerance to higher MTX doses than Caucasian patients, potentially due to population differences in *FPGS* polymorphisms [23]. Therefore, caution is required when generalizing our findings to non-Japanese populations or to treatment settings involving higher MTX doses. Finally, residual bias related to differences in MTX dose distribution between groups may have influenced the results despite stratified randomization.

## Conclusions

In the present study, co-administration of 10 mg folic acid showed little effect on reducing MTX toxicity, suggesting that the routine use of 5 mg folic acid is likely sufficient during MTX therapy in patients with rheumatic diseases. However, in patients who develop fatigue, increasing the dose of folic acid may be a therapeutic option.

## Supplementary Information

Below is the link to the electronic supplementary material.

ESM 1 (DOCX 6.71 MB)

ESM 2 (DOCX 20.4 KB)

## Data Availability

The datasets generated and analyzed in the current study are not publicly available, but are available from the corresponding author upon reasonable request.
