# Supplementary material for: Effect of different folic acid doses on methotrexate-related toxicity and its association with erythrocyte methotrexate-polyglutamates in patients with rheumatic diseases: a single-center exploratory randomized controlled trial
Source: Clin Rheumatol. 2026 Jun 6;45(7):3957–65. doi: 10.1007/s10067-026-08195-8 (PMC13342139; doi:10.1007/s10067-026-08195-8)
Supplement: Supplementary file 1 — (DOCX 6.71 MB) [file 10067_2026_8195_MOESM1_ESM.docx]

**Supplementary Fig. 1 Study design**


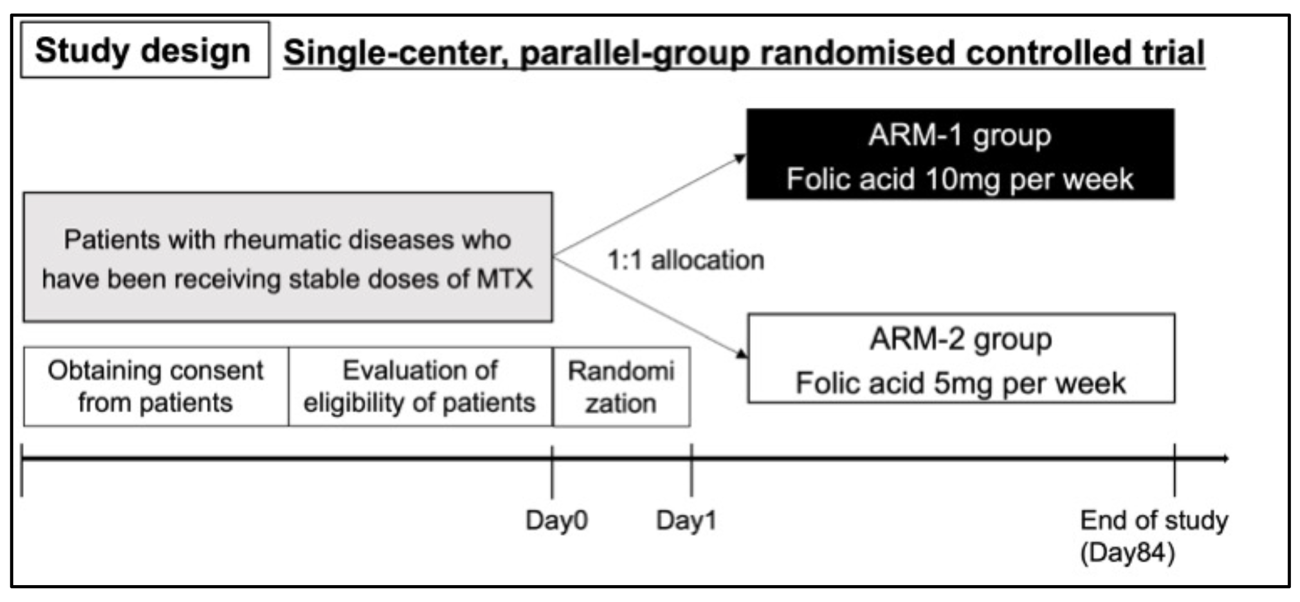


**Supplementary Fig. 2 Baseline MTX dose, dosing schedule, and MTX-PG concentrations.**


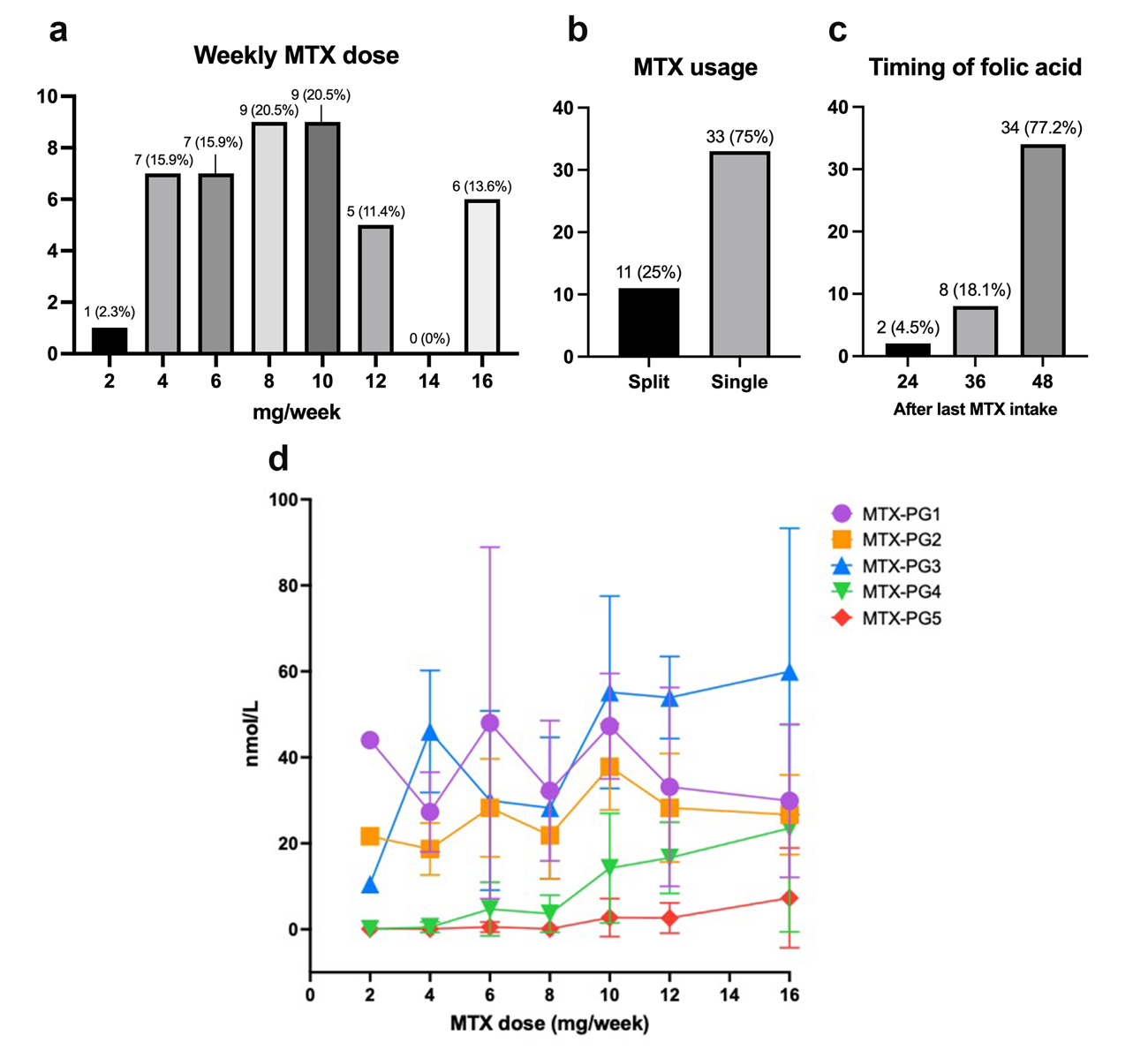


(a–c) Baseline MTX dose and dosing schedule among patients. (d) Association between weekly MTX dose and MTX-PG concentrations.

MTX, methotrexate; MTX-PG, methotrexate-polyglutamate
