# Supplementary material for: Effect of different folic acid doses on methotrexate-related toxicity and its association with erythrocyte methotrexate-polyglutamates in patients with rheumatic diseases: a single-center exploratory randomized controlled trial
Source: Clin Rheumatol. 2026 Jun 6;45(7):3957–65. doi: 10.1007/s10067-026-08195-8 (PMC13342139; doi:10.1007/s10067-026-08195-8)
Supplement: Supplementary file 2 — (DOCX 20.4 KB) [file 10067_2026_8195_MOESM2_ESM.docx]

**Supplementary Table 1. All adverse events not attributable to MTX toxicity during the study period**

|  | **ARM-1 (10mg folic acid)**  **N=22** | **ARM-2 (5mg folic acid)**  **N=22** | **p** |
| --- | --- | --- | --- |
| **Oral herpes infection** | 1 (4.5) | 0 (0) | 1.000 |
| **Acute upper respiratory infection** | 2 (9.1) | 3 (13.6) | 1.000 |
| **Periodontitis** | 1 (4.5) | 0 (0) | 1.000 |
| **Cervical spondylosis** | 0 (0) | 1 (4.5) | 1.000 |
| Variables are described as number (percentage).  Comparison of the data between the ARM-1 vs. ARM-2 was made by chi-square test.  Abbreviations: MTX, methotrexate. | | | |

**Supplementary Table 2. Differences in changes in MTX-PG concentrations at Day84 adjusted for the baseline values between groups**

|  | **Difference in LSM (95% CI), ARM-1 vs ARM-2, p-value** |
| --- | --- |
| **MTX-PG1** | 1.08 (-5.30 to 7.46), p = 0.734 |
| **MTX-PG2** | 0.27 (-3.44 to 3.97), p = 0.886 |
| **MTX-PG1-2** | 0.69 (-8.43 to 9.81), p = 0.880 |
| **MTX-PG3** | 3.55 (-0.52 to 7.62), p = 0.086 |
| **MTX-PG4** | 1.20 (-1.10 to 3.49), p = 0.299 |
| **MTX-PG5** | 0.30 (-0.44 to 1.03), p = 0.417 |
| **MTX-PG3-5** | 4.95 (-1.31 to 11.20), p = 0.118 |
| **MTX-PG4-5** | 1.48 (1.46 to 4.42), p = 0.314 |
| **MTX-PG1-5** | 4.09 (-8.81 to 17.00), p = 0.525 |
| Abbreviations: MTX-PG, methotrexate-polyglutamate; LSM, least-squares mean; CI, confidence interval. | |

**Supplementary Table 3. Randomized controlled trials evaluating different doses of folic acid compared with the present study**

| **First author (year)** | **Patients (Sample size)** | **Comparison of two folic acid regimens** | **Timing of administration of folic acid** | **Outcomes** |
| --- | --- | --- | --- | --- |
| **Morgan SL, et al (1994)** | RA (51)^†^ | Folic acid 27.5 mg per week vs. folic acid 5 mg per week | Both folic acid regimens taken on 5 non-MTX days per week | No differences in benefit or toxicity related to MTX |
| **Dhir V, et al (2015)** | RA (100) | Folic acid 30 mg per week vs. folic acid 10 mg per week | 5 mg folic acid on 6 days per week vs. 5 mg folic acid on 2 days per week, taken only on non-MTX days per week | No differences in benefit or toxicity related to MTX |
| **Stamp LK, et al (2019)** | RA (40) | Folic acid 5 mg per week vs. folic acid 0.8 mg per week | Both folic acid regimens taken on the fourth day after MTX administration | No differences in benefit or toxicity related to MTX |
| **The present trial** | Rheumatic disease (44) ^‡^ | Folic acid 10 mg per week vs. folic acid 5 mg per week | Both folic acid regimens taken on the 24 hours after the latest MTX administration | No differences in benefit or toxicity related to MTX |
| ^†^Of the 79 patients in the study, 28 were allocated to placebo.  ^‡^Of the 44 patients, 37 RA were included.  Abbreviations: RA, rheumatoid arthritis; MTX, methotrexate | | | | |
